# The relationship between the attitude towards physical exercise and the sports consumption demand of the group with a relatively low level of education: a test of the mediating effect of the amount of physical exercise

**DOI:** 10.3389/fpsyg.2025.1626616

**Published:** 2025-08-11

**Authors:** Shunding Hu, Xiaosu Feng

**Affiliations:** School of Physical Education, Liaoning Normal University, Dalian, China

**Keywords:** attitude towards physical exercise, sports consumption demands, amount of physical exercise, lower level of education, China

## Abstract

**Objective:**

Explore the relationship between the attitudes towards physical exercise sports consumption demands among groups with lower education levels, and test the mediating effect of the amount of physical exercise.

**Methods:**

A combination of stratified sampling and convenience sampling was adopted to select 762 individuals who had not received higher education from non-urban areas of Dalian as participants, covering groups with educational backgrounds of junior school or below and senior school (technical secondary school, technical school). The Exercise Attitude Scale, Physical Exercise Rating Scale, and Sports Consumption Demand Scale were used to investigate the participants’ attitudes towards physical exercise, the amount of physical exercise, and sports consumption demands. SPSS 25.0 and AMOS 23.0 software were used to explore the relationships among variables through inter-group comparative analysis, correlation analysis, regression analysis, and mediating effect tests.

**Results:**

There is a significant positive correlation between the attitude towards physical exercise, the amount of physical exercise, and the sports consumption demand (*p* < 0.01). After controlling for relevant confounding factors, the attitude towards physical exercise could positively predict the sports consumption demand (*p* < 0.01). Except that the relationships between behavioral cognition, emotional experience, and the sports consumption demand were not significant (*p* > 0.05), the positive predictive effects of the remaining dimensions were significant (*p* < 0.05). The mediating effect test indicated that the amount of physical exercise played a mediating role between the attitude towards physical exercise and the sports consumption demands (*β* = 0.003, *p* = 0.029).

**Conclusion:**

There is a significant positive correlation between the attitude towards physical exercise and the sports consumption demands among people with lower education levels, and the amount of physical exercise plays a mediating role in the relationship between the two. The conclusions have improved the “attitude → behavior → consumption” theory, providing a basis for the formulation of policies by relevant departments.

## 1 Introduction

Sports consumption is an emerging form of consumption. With the rapid development of the social economy and the continuous improvement of people’s living standards, it has gradually become an indispensable part of people’s lives. Sports consumption plays a role in promoting the development of the national sports industry and the growth of the social economy (Li and Ling, 2025; Li and Yue, 2025). Sports consumption originates from individuals’ sports consumption demands. When residents have a strong desire for activities such as fitness workouts, sports event viewing, and sports training, consumption behaviors like purchasing sports equipment, signing up for fitness courses, and snapping up event tickets will follow. Conversely, if demand is sluggish, even with a wide variety of sports products and services available in the market, the vitality of consumption is difficult to stimulate. Therefore, how to increase residents’ sports consumption demands has gradually become a topic of concern for researchers.

In China, the group with a low level of education accounts for a considerable proportion of society (Zhang, 2014). Their sports consumption demands are an important part of the sports market. However, due to their relatively low level of education, there may be some unique features in aspects such as their sports consumption concepts and behaviors (Lu, 2022). For example, in terms of consumption concepts, they often place more emphasis on practicality and cost-effectiveness, and have a high sensitivity to prices. They tend to choose inexpensive basic sports equipment and have a low acceptance of sports products with high brand premiums. In the selection of sports events, they are more inclined to traditional sports that are free or low-cost, such as running and square dancing, and show little interest in emerging sports events that require payment to participate. In addition, in terms of consumption behaviors, the limitations of time and space are also quite obvious. Constrained by high-intensity work and limited leisure time, their frequency of participating in sports consumption is relatively low. At the same time, the lack of complete sports facilities around their place of residence further hinders their sports consumption behaviors. Therefore, studying the sports consumption demands of the group with a low level of education can help the sports industry better meet the needs of different groups, and promote the innovation and optimization of sports products and services. Moreover, it is also helpful to increase the sports participation rate of the group with a low level of education, improve their quality of life, and promote the development of the national fitness campaign.

The attitude towards physical exercise is a comprehensive manifestation of an individual’s cognitive evaluation, emotional experience, and behavioral intention towards physical exercise. There is a close connection between the attitude towards physical exercise and the demand for sports consumption. Two studies (Chen et al., 2024; Zhang et al., 2024) on urban residents in China have found that there is a significant positive correlation between the attitude towards physical exercise and the demand for sports consumption. In addition, a study (Xu, 2020) on college students in Shanghai, China, has found that after controlling for relevant confounding factors, there is a significant positive correlation between college students’ attitude towards physical exercise and their demand for sports consumption. However, current research on the group with lower educational attainment is relatively scarce, and it remains unclear whether the relationship between physical exercise attitude and sports consumption demand in this group is consistent with that found in Chinese urban residents (Chen et al., 2024; Zhang et al., 2024) and college students (Xu, 2020). Given the large population size of the lower-educated group, it is necessary to conduct research on this topic. Based on previous studies, this research proposes the research hypothesis H1:

*H1:* There is a significant positive correlation between the attitude toward physical exercise and the demand for sports consumption among the lower-educated group.

The amount of physical exercise directly reflects the degree of an individual’s participation in physical activities, and there is also a close relationship between it and the demand for sports consumption (Lu, 2024). Generally, the greater the amount of physical exercise, the higher the demand for sports goods, venue facilities, etc. The Theory of Planned Behavior holds that behavioral intention is a direct factor influencing behavior, and behavioral intention is affected by three factors: attitude, subjective norm, and perceived behavioral control (Ajzen, 1991). In the field of physical exercise, an individual’s positive attitude towards physical exercise will prompt them to generate the behavioral intention to participate in physical exercise, which in turn leads to the occurrence and maintenance of physical exercise behavior (Godin et al., 1993). In studies on low-income groups, researchers (Armitage and Sprigg, 2010; Meyer, 2024; Zhang, 2018) have explored the practicality of the Theory of Planned Behavior and found that behavioral intention can significantly predict the physical exercise behavior of low-income groups. Therefore, the above-mentioned studies suggest that there is a positive correlation between attitudes toward physical exercise and the amount of physical exercise behavior.

In addition, the consumer behavior theory emphasizes that when making consumption decisions, consumers are influenced by many factors, including personal factors, psychological factors, and social factors (Katona, 1968; Saeed, 2019). In sports consumption, researchers (Chen et al., 2008; Chen et al., 2024; Zhang et al., 2024) have further confirmed that physical exercise is an important influencing factor for sports consumption demand. Chen et al. (2008) investigated the exercise stages and sports consumption needs of 587 intellectuals. By analyzing the sports consumption demands of different stage groups, it was shown that during the positive transformation of physical exercise behavior, the amount of sports consumption demand showed a gradually increasing trend. In addition, two studies based on urban residents in China (Chen et al., 2024; Zhang et al., 2024) also found that there is a potential relationship between the amount of physical exercise and the demand for sports consumption. Based on this, this study proposes the research hypothesis H2:

*H2:* The amount of physical exercise plays a mediating role in the relationship between attitudes toward physical exercise and sports consumption demand.

Given the current research’s insufficient attention to groups with lower educational attainment, this study focuses on the relationship between attitudes toward physical exercise and sports consumption demand within this population, and further explores the mediating role of amount of physical exercise in the above-mentioned relationship. Through this study, it is expected to provide references for formulating effective marketing strategies and policy measures, so as to better meet the sports consumption needs of groups with low educational levels and promote the healthy development of the sports industry.

## 2 Methods

### 2.1 Participants

The group with a low educational background in this study generally refers to those who have not completed higher education, specifically including individuals with an educational attainment of junior high school or below, as well as those with a senior high school (technical secondary school, technical school) education. This group may have some characteristics different from those of the group with a high educational background in terms of socioeconomic status, career development, and lifestyle, etc., and their sports consumption needs may also exhibit a unique pattern.

This study collected data from July to August 2024 in the non-urban areas of Dalian City, China (Lüshunkou District, Pulandian District, Zhuanghe City, and Wafangdian City), with 200 questionnaires distributed in each urban area. This study adopted a combined method of convenience sampling and stratified sampling to distribute questionnaires. First, the survey in the sample cities was mainly targeted at people with an educational attainment below senior high school (including technical secondary schools and technical schools). To facilitate contact with this group, the questionnaires were mainly distributed in places such as communities, factory entrances, and vegetable markets. Second, during the questionnaire distribution process, stratification was carried out according to gender to ensure, as much as possible, the gender balance of the respondents.

This study established the following questionnaire elimination criteria: (1) Large-area missing answers in the questionnaire, such as entire pages left unanswered due to forgetting to turn pages; (2) Random filling of the questionnaire, such as respondents basically checking one option for the entire questionnaire or alternating between multiple options; (3) Random filling of basic information, such as height and weight significantly deviating from normal values. For questionnaires with a small number of missing or incorrectly filled answers, the researchers dealt with them as missing values and filled them using the method of linear interpolation. After eliminating the invalid questionnaires, a total of 762 valid questionnaires were obtained in this study. This study was conducted in accordance with the Declaration of Helsinki. All participants gave their informed consent, and this study was approved by the Ethics Committee of Liaoning Normal University.

### 2.2 Tools

#### 2.2.1 Exercise attitude scale

In the study of attitudes towards physical exercise, this study selected the *Exercise Attitude Scale* developed by Mao (2003) as the measurement tool. This scale constructs a multi-dimensional evaluation system, covering eight core dimensions: behavioral attitude, goal attitude, behavioral cognition, behavioral habit, behavioral intention, emotional experience, sense of behavioral control, and subjective criteria, with a total of 70 items set. The scale adopts a 5-point scoring method, assigning scores from 1 to 5 in sequence from “strongly disagree” to “strongly agree.” The higher the score, the more positive the residents’ attitude towards exercise. From the results of the reliability and validity tests, the scale demonstrates good measurement quality. In terms of reliability, except that the Cronbach’s *α* coefficient of the subjective criteria dimension exceeds 0.6, the Cronbach’s *α* coefficients of the remaining dimensions all reach above 0.8, indicating a high level of internal consistency of the scale. In the validity test, the model fitting indexes perform well. The value of *χ*^2^/*df* is 3.66, which is less than the critical value of 5; the value of NNFI and CFI are close to or greater than 0.90; and the value of RMSEA is less than 0.10. This series of data shows that the scale has good construct validity and can effectively and accurately measure the residents’ attitudes towards physical exercise.

#### 2.2.2 Physical exercise rating scale (PERS-3)

In the survey of the physical exercise level of residents, this study employed the *Physical Exercise Rating Scale (PERS-3)* revised by Liang (1994). This scale focuses on three key dimensions: the intensity, duration, and frequency of physical exercise to evaluate the residents’ physical exercise level. In terms of specific classification, both the intensity and duration of physical exercise are divided into five grades ranging from 1 to 5, while the frequency of physical exercise is divided into five grades ranging from 0 to 4. After testing, the test–retest reliability of this questionnaire reaches 0.82, indicating a relatively high level of reliability. The calculation of the physical exercise level is based on a specific formula, that is, it is obtained by multiplying the intensity, duration, and frequency of physical exercise together (Physical Exercise Level = Intensity of Physical Exercise × Duration of Physical Exercise × Frequency of Physical Exercise). Based on this, the physical activity level is classified into different levels: when the calculation result is less than or equal to 19 points, it is determined as a small amount of exercise; when it is in the range of 20 to 42 points, it is defined as a moderate amount of exercise; and when the score reaches 43 points or more, it is classified as a large amount of exercise (Liang, 1994). The five levels of physical exercise intensity are described in detail to help participants understand their exercise intensity: (1) Light exercise, such as walking, doing radio calisthenics, etc. (2) Low-intensity and non-strenuous exercise, such as recreational volleyball, yoga, jogging, tai chi, etc. (3) Moderate-intensity and relatively intense sustained exercise, such as cycling, running, table tennis, weightlifting in the gym, etc. (4) High-intensity exercise with rapid breathing and heavy sweating, but not sustained (e.g., badminton, basketball, tennis, football, etc.). (5) High-intensity and sustained exercise with rapid breathing and heavy sweating (e.g., sprinting, complete aerobics routines, swimming, etc.). In addition, researchers provided guidance on questionnaire responses. If participants did not fully understand the items, detailed explanations were given to ensure measurement validity.

#### 2.2.3 Sports consumption demand scale

In order to explore the status of residents’ sports consumption demand, this study selected the *Sports Consumption Demand Scale* developed by Chen et al. (2008) as the survey tool. This scale uses the Likert 5-point rating system and comprehensively evaluates residents’ sports consumption demand starting from 10 items. Its evaluation framework covers four major dimensions: physical goods demand, information materials demand, viewing demand, and participation demand. In terms of the quality inspection of the scale, all indicators perform excellently. After testing, the Cronbach’s *α* coefficients of the four dimensions of this scale are all higher than 0.7, indicating good internal consistency. At the same time, the results of the exploratory factor analysis and confirmatory factor analysis show that the model has an ideal fit, fully demonstrating that this scale has reliable construct validity and can scientifically and effectively measure the status of residents’ sports consumption demand.

#### 2.2.4 Self-compiled demographic information questionnaire

In this study, a self-compiled demographic information questionnaire was used to survey the participants’ basic information such as age, gender, height, weight, educational attainment, etc. In this study, the Body Mass Index (BMI) of the participants was calculated according to the BMI calculation formula [weight (in kilograms) divided by the square of height (in meters)].

### 2.3 Data collection process

During the data collection process, first of all, the investigators were uniformly trained to make them familiar with the content of the questionnaire, the survey process, and the matters needing attention, so as to ensure the standardization and consistency of the survey. The investigators included 2 postgraduate students majoring in sports, who had good communication skills and a strong sense of responsibility. In the selected places, the investigators distributed the questionnaires to the subjects and explained on the spot the filling methods and matters needing attention of the questionnaires, encouraging the subjects to fill them out truthfully. For some participants with a relatively low educational level who might have difficulties in understanding, the investigators patiently explained the meanings of the questions to ensure that the subjects could answer accurately. After the questionnaires were filled out, the investigators collected them on the spot, and conducted a preliminary screening of the questionnaires that were incompletely filled out or obviously illogical, and carried out supplementary investigations. During the data collection process, the data quality was strictly controlled. The collected questionnaires were reviewed and sorted out every day, and problems were discovered and solved in a timely manner. For the data in question, verification was carried out by means of telephone follow-up or another on-site investigation.

### 2.4 Statistical methods

This study uses SPSS 25.0 and AMOS 23.0 software to process and statistically analyze the data of the recovered questionnaires. A one-sample Kolmogorov–Smirnov test, combined with the kurtosis and skewness of the data, is used for the normality test. It is found that the data roughly follow a normal distribution, so parametric tests (independent samples *t*-test and one-way analysis of variance) are adopted to conduct inter-group comparative analysis of demographic variables. In the independent samples *t*-test, Levene’s test is used for the homogeneity of variance test. If the variances are homogeneous, the results assuming equal variances are adopted for analysis; if the variances are heterogeneous, the results not assuming equal variances are used for analysis. In the one-way analysis of variance, Levene’s test is employed for the homogeneity of variance test. When the variances are homogeneous, the LSD method is used for intergroup comparative analysis; when the variances are heterogeneous, the Tamhane’s method is applied for intergroup comparative analysis.

Pearson correlation analysis is used to explore the correlations among the attitude towards physical exercise, the amount of physical exercise, and the demand for sports consumption. In addition, this study constructs a multiple linear regression model. After controlling relevant confounding factors, it explores the relationship between the attitude towards physical exercise and the demand for sports consumption. In the analysis, the Variance Inflation Factor (VIF) was used to diagnose the collinearity of independent variables. Finally, taking the demand for sports consumption as the dependent variable, the attitude towards physical exercise as the independent variable, and the amount of physical exercise as the mediating variable, this study explores the mediating effect of the amount of physical exercise between the attitude towards physical exercise and the demand for sports consumption. The values of CMIN/DF, RMSEA, NFI, RFI, IFI, TLI, and CFI are used for the model fit test. If the CMIN/DF value is between 1 and 3, it indicates a good model fit; if the RMSEA value is between 0.08 and 0.10, it represents a general fit, if it is between 0.05 and 0.08, it represents a reasonable fit, and if it is less than 0.05, it indicates a very high fit. The values of NFI, RFI, IFI, TLI, and CFI mostly range from 0 to 1, and the closer they are to 1, the better the model fit. Among them, the values of TLI, CFI, and IFI may be greater than 1 (Wu, 2010). The significance level of all statistical methods involved in this study is defined as *α* = 0.05.

## 3 Results

### 3.1 The basic information of the participants

Among the 762 participants included, the majority were from the groups of those under 20 years old (34.1%), those aged 21 to 30 years old (26.5%), and those aged 31 to 40 years old (21.8%). In terms of gender, males accounted for 49.6% and females accounted for 50.4%. In terms of educational attainment, those with a junior school education or below accounted for 11.8%, and those with a senior school (technical secondary school, technical school) education accounted for 88.2%. In addition, the average BMI of the participants was (22.61 ± 3.66) kg/m^2^, which was within the normal weight range overall. The average score of the attitude towards physical exercise was (261.26 ± 55.82) points, which was at a moderately high level; the average score of the demand for sports consumption was (41.27 ± 27.23) points, which were at a relatively high level; and the average score of the amount of physical exercise was (37.17 ± 8.37) points, which was at a moderate level. Details of the participants are shown in Table 1.

**Table 1:** The basic information of the participants.

Variables	Freq.	%
Age		
Under 20	260	34.1
21 ~ 30	202	26.5
31 ~ 40	166	21.8
41 ~ 50	94	12.3
Over 51	40	5.2
Gender		
Male	378	49.6
Female	384	50.4
Education level		
Junior school and below	90	11.8
Senior school	672	88.2

Variables	M	SD
BMI	22.61	3.66
Attitude towards physical exercise	261.26	55.82
Sports consumption demand	41.27	27.23
Amount of physical exercise	37.17	8.37

### 3.2 Comparative analysis among groups of the participants’ attitudes towards physical exercise, the amount of physical exercise, and the demand for sports consumption

**Figure 1:**
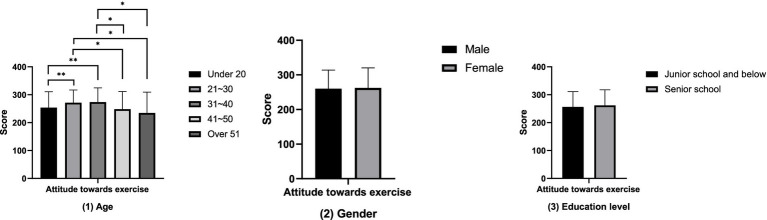
Results of the comparative analysis of attitudes towards physical exercise between groups (*p < 0.05; **p < 0.01).

**Figure 2:**
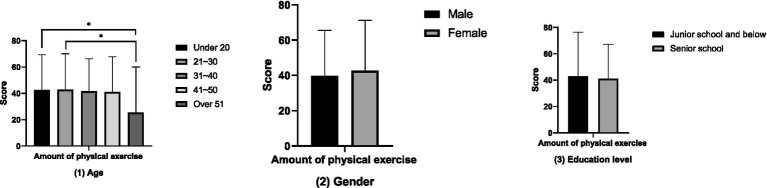
Results of the comparative analysis of amount of physical exercise between groups (*p < 0.05; **p < 0.01).

**Figure 3:**
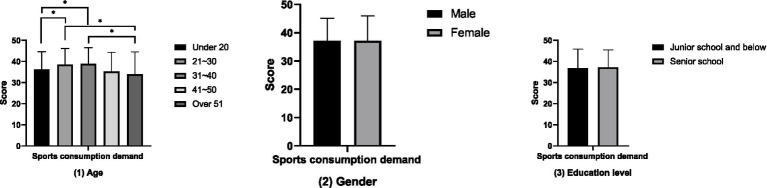
Results of the comparative analysis of sports consumption demand between groups (*p < 0.05; **p < 0.01).

The results of the comparative analysis of attitudes towards physical exercise between groups (Figure 1) show that the attitudes towards physical exercise of the groups aged 21–30 and 31–40 are significantly higher than those of the groups under 20 years old, 41–50 years old, and over 51 years old (*p* < 0.05). However, no differences in attitudes towards physical exercise have been found in terms of gender and educational attainment (*p* > 0.05). The results of the between-group comparative analysis of the amount of physical exercise (Figure 2) showed that the amount of physical exercise of the groups under 20 years old and those aged 21 to 30 was significantly higher than that of the group over 51 years old (*p* < 0.01). However, this study did not find any differences in the amount of physical exercise in terms of gender and educational attainment (*p* > 0.05). The results of the between-group comparative analysis of sports consumption demand (Figure 3) show that the sports consumption demand of the groups aged 21 to 30 and 31 to 40 is significantly higher than that of the groups under 20 years old and over 51 years old (*p* < 0.05). However, this study has not found significant differences in sports consumption demand in terms of gender and educational attainment (*p* > 0.05).

### 3.3 The correlations among the attitude towards physical exercise, the amount of physical exercise, and the demand for sports consumption

The results of the correlation analysis of the attitude towards physical exercise, the amount of physical exercise, and the demand for sports consumption (Table 2) show that there is a significant positive correlation between the demand for sports consumption and the amount of physical exercise (*r* = 0.226, *p* < 0.01), and there is a significant positive correlation with the demand for sports consumption (*r* = 0.795, *p* < 0.01); there is a significant positive correlation between the amount of physical exercise and the demand for sports consumption (*r* = 0.187, *p* < 0.01). In addition, there are significant positive correlations between the sub-dimensions of the attitude towards physical exercise, the amount of physical exercise, and the sub-dimensions of the demand for sports consumption (*p* < 0.01). In conclusion, there are connections among the attitude towards physical exercise, the amount of physical exercise, and the demand for sports consumption, which provides a basis for our subsequent analysis.

**Table 2:** The results of the correlation analysis of the attitude towards physical exercise, the amount of physical exercise, and the demand for sports consumption.

Variables	1	2	3	4	5	6	7	8	9	10	11	12	13	14	15
1	1.000	0.951**	0.959**	0.934**	0.960**	0.934**	0.959**	0.935**	0.934**	0.226**	0.910**	0.795**	0.822**	0.744**	0.795**
2	0.951**	1.000	0.905**	0.868**	0.898**	0.872**	0.893**	0.886**	0.878**	0.211**	0.866**	0.757**	0.777**	0.713**	0.759**
3	0.959**	0.905**	1.000	0.878**	0.899**	0.872**	0.908**	0.873**	0.886**	0.192**	0.875**	0.775**	0.793**	0.715**	0.750**
4	0.934**	0.868**	0.878**	1.000	0.890**	0.852**	0.874**	0.864**	0.869**	0.215**	0.839**	0.733**	0.754**	0.687**	0.737**
5	0.960**	0.898**	0.899**	0.890**	1.000	0.889**	0.918**	0.876**	0.882**	0.220**	0.867**	0.750**	0.788**	0.704**	0.769**
6	0.934**	0.872**	0.872**	0.852**	0.889**	1.000	0.890**	0.854**	0.852**	0.228**	0.847**	0.737**	0.769**	0.698**	0.733**
7	0.959**	0.893**	0.908**	0.874**	0.918**	0.890**	1.000	0.885**	0.871**	0.210**	0.866**	0.745**	0.778**	0.716**	0.771**
8	0.935**	0.886**	0.873**	0.864**	0.876**	0.854**	0.885**	1.000	0.855**	0.221**	0.861**	0.755**	0.780**	0.698**	0.751**
9	0.934**	0.878**	0.886**	0.869**	0.882**	0.852**	0.871**	0.855**	1.000	0.225**	0.864**	0.766**	0.783**	0.695**	0.748**
10	0.226**	0.211**	0.192**	0.215**	0.220**	0.228**	0.210**	0.221**	0.225**	1.000	0.241**	0.212**	0.243**	0.183**	0.187**
11	0.910**	0.866**	0.875**	0.839**	0.867**	0.847**	0.866**	0.861**	0.864**	0.241**	1.000	0.895**	0.893**	0.823**	0.854**
12	0.795**	0.757**	0.775**	0.733**	0.750**	0.737**	0.745**	0.755**	0.766**	0.212**	0.895**	1.000	0.726**	0.645**	0.688**
12	0.822**	0.777**	0.793**	0.754**	0.788**	0.769**	0.778**	0.780**	0.783**	0.243**	0.893**	0.726**	1.000	0.630**	0.688**
14	0.744**	0.713**	0.715**	0.687**	0.704**	0.698**	0.716**	0.698**	0.695**	0.183**	0.823**	0.645**	0.630**	1.000	0.644**
15	0.795**	0.759**	0.750**	0.737**	0.769**	0.733**	0.771**	0.751**	0.748**	0.187**	0.854**	0.688**	0.688**	0.644**	1.000

1. Behavior attitude; 2. Goal attitude; 3. Behavior cognition; 4. Behavior habit; 5. Behavior intention; 6. Emotional experience; 7. Sense of behavior control; 8. Subjective standard; 9. Attitude towards physical exercise; 10. Amount of physical exercise; 11. Participatory needs; 12. Physical object needs; 13. Information needs; 14. Watching needs; 15. Sports consumption needs. **indicates *p* < 0.01.

### 3.4 The relationship between the attitude towards physical exercise and the demand for sports consumption

Before the analysis, this study conducted collinearity diagnosis and found that in the regression model of physical exercise attitude and sports consumption demand, the VIF of physical exercise attitude was 1.008, so there was no collinearity; in the regression models constructed by each sub-dimension of physical exercise attitude and sports consumption demand, the VIF of the sub-dimensions was between 1.003 and 1.014, so there was no collinearity. After controlling for relevant confounding variables, the relationship between the attitude towards physical exercise and the demand for sports consumption was explored (Table 3). It was found that the attitude towards physical exercise could positively predict the demand for sports consumption (*β* = 0.136, 95%CI = 0.131–0.140, Wald *χ*^2^ = 3571.313, *p* = 0.000). That is, for every one-unit increase in the attitude towards physical exercise, the demand for sports consumption increases by 0.136 units. In addition, it was further found that the behavioral attitude, target attitude, behavioral habits, behavioral intention, sense of behavioral control, and subjective criteria could positively predict the demand for sports consumption (*p* < 0.05). However, the relationships between behavioral cognition, emotional experience, and the demand for sports consumption were not significant (*p* > 0.05). In terms of control variables, there was a significant positive correlation between BMI and the demand for sports consumption (*p* < 0.01), while there were no significant correlations between age, gender, educational attainment, and the demand for sports consumption (*p* > 0.05).

**Table 3:** The results of the multiple linear regression analysis of the attitude towards physical exercise and the demand for sports consumption.

Variables	*β*	95%CI	Wald *χ*^2^	*p*
Attitude towards physical exercise	0.136	0.131, 0.140	3571.313	0.000
Behavior attitude	0.137	0.038, 0.235	7.427	0.006
Goal attitude	0.174	0.101, 0.247	21.703	0.000
Behavior cognition	−0.025	−0.128, 0.078	0.226	0.634
Behavior habit	0.100	0.013, 0.188	5.042	0.025
Behavior intention	0.130	0.039, 0.221	7.887	0.005
Emotional experience	0.067	−0.022, 0.156	2.175	0.140
Sense of behavior control	0.216	0.123, 0.309	20.672	0.000
Subjective standard	0.295	0.193, 0.398	32.146	0.000
Age				
Under 20	−0.220	−1.362, 0.923	0.142	0.706
21 ~ 30	−0.100	−1.276, 1.076	0.028	0.868
31 ~ 40	−0.294	−1.491, 0.904	0.231	0.631
41 ~ 50	−0.504	−1.769, 0.762	0.609	0.435
Gender				
Male	0.120	−0.370, 0.610	0.231	0.631
Education level				
Junior school and below	0.347	−0.488, 1.183	0.664	0.415
BMI	0.192	0.125, 0.259	31.347	0.000
	−0.238	−1.372, 0.895	0.170	0.680
	−0.128	−1.292, 1.036	0.046	0.830
	−0.200	−1.384, 0.985	0.109	0.741
	−0.417	−1.666, 0.832	0.428	0.513
	0.175	−0.317, 0.667	0.487	0.485
	0.405	−0.423, 1.232	0.917	0.338
	0.202	0.135, 0.270	36.649	0.000

### 3.5 Test of the mediating effect of the amount of physical exercise between the attitude towards physical exercise and the demand for sports consumption

The test results of the model goodness of fit (Table 4) show that CMIN/DF = 2.325, RMSEA = 0.042, and the values of NFI, RFI, IFI, TLI, and CFI are 0.989, 0.986, 0.994, 0.992, and 0.994, respectively. Therefore, the overall model fitting effect is good. The study uses maximum likelihood estimation for the coefficient values of each path. The results (Table 5) show that the coefficient values of Path 1, Path 2, and Path 3 all reach statistical significance (*p* < 0.05), indicating that the path through which the attitude towards physical exercise affects the demand for sports consumption through the amount of physical exercise activities is valid.

**Table 4:** The test results of the goodness of fit of the structural equation model.

Statistic	Critical value	Test result	Judgment of model goodness of fit
CMIN/DF	<3	2.325	Meet
RMSEA	<0.05	0.042	Meet
NFI	>0.9	0.989	Meet
RFI	>0.9	0.986	Meet
IFI	>0.9	0.994	Meet
TLI	>0.9	0.992	Meet
CFI	>0.9	0.994	Meet

**Table 5:** Path analysis coefficient table.

Path		β	SE	CR	*P*
1	Amount of physical exercise <− Attitude towards physical exercise	0.925	0.145	6.375	<0.001
2	Sports consumption needs <− Amount of physical exercise	0.003	0.001	2.183	0.029
3	Sports consumption needs <− Attitude towards physical exercise	0.328	0.010	33.557	<0.001

**Figure 4:**
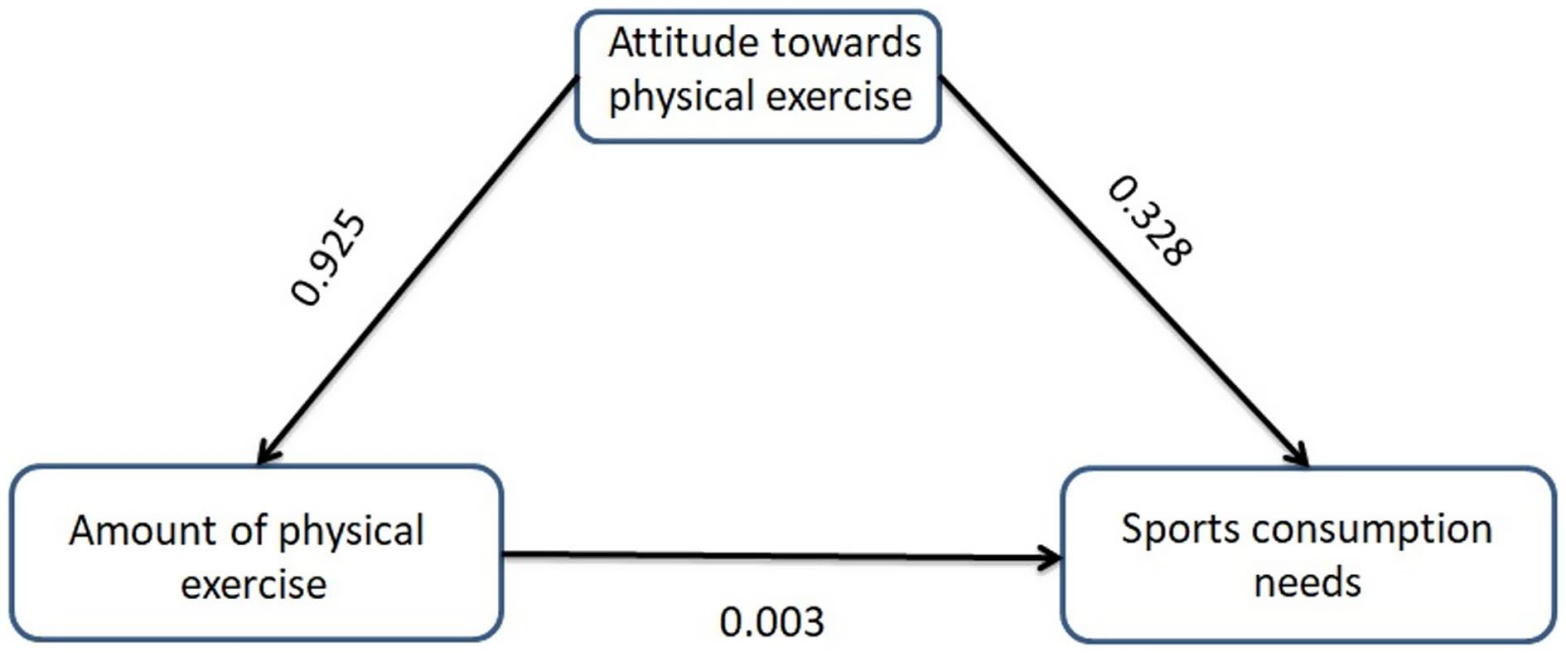
Mediating effect diagram.

According to the mediating effect diagram (Figure 4), the direct effect of physical exercise attitude on sports consumption demand is 0.328. The indirect effect of physical exercise attitude on sports consumption demand through physical exercise volume is 0.925 × 0.003 = 0.003. Therefore, the total effect of physical exercise attitude on sports consumption demand is 0.328 + 0.003 = 0.331.

## 4 Discussion

### 4.1 Discussion of the main results

This study further refined the research on the group with a low educational level, and explored in greater depth the relationships among the attitude towards physical exercise, the amount of physical exercise, and the demand for sports consumption of this group. The results of this study found that there is a significant positive correlation between attitudes towards physical exercise and the sports consumption demands of groups with lower education levels. Moreover, the amount of physical exercise has a significant mediating effect between the two. These findings are similar to those of previous studies on urban residents in China (Chen et al., 2024; Zhang et al., 2024). This suggests that by enhancing the attitudes towards physical exercise among groups with lower education levels and increasing their actual physical exercise volume, their sports consumption demands can be enhanced.

This study explains why the research findings are similar to previous studies on urban residents (Chen et al., 2024; Zhang et al., 2024) from three dimensions of group commonalities and theoretical support. Firstly, with the acceleration of urbanization, lower-educated groups also enjoy similar living environments (such as the popularization of community fitness facilities and commercial fitness institutions) and consumption scenarios (such as online sports product purchases and offline fitness activities). In addition, as residents’ attention to health generally increases, the demand for “improving health through exercise” regardless of educational level will be transformed into actual demand for sports consumption. Secondly, there is a social learning effect in consumption behavior. According to the social cognitive theory, an individual’s behavior is formed through observing, imitating, and learning from others (Bandura, 2001). Meanwhile, an individual’s cognition and self-efficacy also significantly influence behavior (Bandura, 2001). At the level of sports consumption, low-educated residents may observe groups around them who often participate in sports activities or engage in sports consumption and imitate their behaviors. If low-educated groups have good experiences in physical exercise and enhance their self-efficacy, they are more likely to continue participating in physical exercise and increase sports consumption. Therefore, there may be *a priori* connections between low-educated groups’ attitudes toward physical exercise and their demand for sports consumption. In summary, the commonalities in the research findings mainly stem from the universality of the “behavior-consumption” logic. Whether for low-educated groups or ordinary urban residents, their sports consumption behaviors follow the basic logic of “attitudes driving behavior, and behavior generating demand,” and the homogenization of urban living environments and consumption concepts further strengthens this association.

The results of this study found a significant positive correlation between the physical exercise attitude and sports consumption demand among the low-educated group. This result indicates that a positive attitude towards physical exercise can significantly stimulate the willingness of this group to participate in sports consumption. The Howard-Sheth model is a typical consumer behavior theory model, which expounds the influencing factors of individual consumption behavior from four aspects: internal factors, external factors, stimulating factors, and purchasing experience (Farley and Ring, 1970; Hunt and Pappas, 1972). Wang (2018) summarized the factors of sports consumption, among which sports demand, sports motivation, sports attitude, etc. are relatively important internal factors. Therefore, the attitude towards physical exercise is closely related to the demand for sports consumption. In addition, dimensions such as behavioral attitude, goal attitude, behavioral habits, behavioral intention, sense of behavioral control, and subjective norms have a positive predictive effect on the demand for sports consumption, which means that when an individual forms a positive cognitive evaluation of physical exercise (such as recognizing the health value of exercise), sets clear exercise goals (such as losing weight and enhancing physical fitness), develops regular exercise habits, generates a strong willingness to exercise, perceives their own control over exercise behavior, and follows the social norms’ expectations of physical exercise, they are more inclined to engage in sports consumption. However, the relationship between the dimensions of behavioral cognition and emotional experience and the demand for sports consumption is not significant. The reasons for this may be that the low-educated group tends to view physical exercise from a “pragmatic” and “result-oriented” perspective rather than focusing on emotional or cognitive experiences during exercise. For example, they care more about specific outcomes such as “whether exercise can help lose weight” or “improve health” rather than abstract feelings like “whether exercise is enjoyable” or “whether it brings psychological relaxation.” In addition, the behavioral cognition dimension (such as rational cognition of exercise and understanding of exercise principles) requires a certain level of knowledge reserve. Due to limited knowledge acquisition ability, the low-educated group may only stay on the surface of “whether it is effective” in their cognition of exercise, rather than deeply understanding the behavioral logic, so the influence of this dimension on consumption demand is not significant.

The results of this study found that the amount of physical exercise plays a mediating role between physical exercise attitude and physical exercise consumption demand. This result is in line with the expectations of the Theory of Planned Behavior (Meyer, 2024) and the Consumer Behavior Theory (Han, 2021). Attitude and behavior are interconnected. The results of this study found that the amount of physical exercise plays a mediating role between physical exercise attitude and physical exercise consumption demand (Myers, 2006). In addition, the Theory of Planned Behavior points to exercise intention as the origin of exercise behavior, that is, the degree to which an individual subjectively hopes to actively participate in physical exercise (Yang, 2012), which is closely related to the attitude towards physical exercise. Therefore, there is a close connection between the attitude towards physical exercise and the physical exercise behavior. By promoting the attitude of residents towards physical exercise, their exercise intention can be strengthened, and the amount of their physical activity can be increased. Moreover, physical exercise can generate the exercisers’ demands for sports equipment, exercise materials, fitness equipment, etc. Therefore, the amount of physical exercise plays a mediating role between the attitude towards physical exercise and the demand for sports consumption.

In addition, this study also found that the 21 ~ 40 age group has higher physical exercise attitudes and sports consumption needs. Firstly, the 21 ~ 40 age group belongs to early adulthood to pre-middle age, with stronger adaptability and experience in sports, thus forming a positive exercise attitude. Secondly, the income level of the 21 ~ 40 age group is significantly higher than that of adolescents, and the proportion of disposable income used for “self-investment” (such as purchasing smart sports watches, fitness personal training courses, high-end sportswear, etc.) has increased, providing the economic foundation for sports consumption. Furthermore, compared with the older generation’s “thrifty consumption” concept, this group more strongly agrees with the idea that “health is an investment” and is willing to pay for sports experiences.

### 4.2 The theoretical significance and practical implications of this study

From a theoretical perspective, this study has refined the theoretical model of sports consumption behavior. Previous studies have explored sports consumption behavior, but there is a relative lack of research on the specific group with a lower educational level. This study incorporates this group into the research scope, conducts an in-depth analysis of the relationships among the attitude towards physical exercise, the amount of physical exercise, and the demand for sports consumption, filling the gap in the research on this group within the theoretical model and making the theoretical model of sports consumption behavior more comprehensive and complete. Secondly, this study has revealed the internal mechanism of the relationship between the attitude towards physical exercise and the demand for sports consumption. Empirical analysis has confirmed that: the attitude towards physical exercise not only has a significant positive correlation with sports consumption demand, but also indirectly relates to sports consumption demand through the mediating variable of physical exercise volume. This discovery has deepened our understanding of the internal logic of sports consumption behavior and provided new perspectives and ideas for subsequent studies.

The results of this study provide a clear direction for promoting the sports consumption of the group with a lower educational level. To increase the sports consumption demand of this group, efforts can be made to improve their attitude towards physical exercise. The government should increase its investment in the construction of sports infrastructure, especially in communities and townships where the group with a lower educational level is concentrated. More free or low-cost sports venues, fitness squares and other facilities should be built to lower the threshold for the group with a lower educational level to participate in physical exercise, increase their amount of physical exercise, and thus promote sports consumption. In addition, this study suggests that at the community level, low-cost and highly accessible fitness programs should be launched, such as evening square dance courses and free community morning running groups, to lower the participation threshold for the less educated population. At the enterprise level, it is recommended to develop “durable” affordable sports equipment (such as wear-resistant running shoes priced below $15) and avoid excessive marketing of emotionally premium products, so as to meet basic sports needs with high cost performance.

### 4.3 The limitations of this study

Although this study has, to a certain extent, confirmed the relationship among the attitude towards physical exercise, the amount of behavioral activities, and the sports consumption demand of the group with a low level of education, which has guiding significance for promoting the sports consumption of this group and formulating relevant policies for the sports industry. However, there are the following limitations in this study.

Firstly, the sample of this study only selects the group with a low level of education in the non-urban areas of Dalian. The regional representativeness of the sample and the diversity of the socioeconomic background are relatively insufficient, which may affect the universality of the research conclusions. Future studies can expand the sample range to cover the group with a low level of education in different regions and occupations, and further verify the research conclusions.

Secondly, this study uses cross-sectional data for analysis, making it difficult to reveal the dynamic change relationship among variables. Subsequent studies can adopt longitudinal follow-up research to deeply explore the causal relationship and development and change rules among the attitude towards physical exercise, the amount of exercise, and the sports consumption demand.

Thirdly, this study has not controlled for confounding factors such as income and occupation type, which may lead to instability in the research results. Therefore, it is recommended that future studies further control relevant potential confounding factors to improve the accuracy of research findings.

Finally, the participants included in this study cover educated groups at different levels, including moderately educated groups (such as senior school groups) and very low-educated groups (such as junior school and below groups). In particular, this study included more senior school groups, so the results may be more inclined to this group. Therefore, follow-up studies need to further include more groups with lower educational levels, and through subgroup analysis, explore the heterogeneity of the relationship among physical exercise attitude, physical exercise amount, and sports consumption demand in different educated groups.

## 5 Conclusion

This study focuses on the relationship among the physical exercise attitude, exercise amount, and sports consumption demand of groups with lower educational attainment. It is found that there is a positive correlation between the physical exercise attitude and sports consumption demand in these groups, and the amount of physical exercise plays a mediating role in the relationship between physical exercise attitude and sports consumption demand. This finding verifies the theoretical logic of “attitude→ behavior→ consumption demand,” which is in line with the expectations of the Theory of Planned Behavior and Consumer Behavior Theory. Based on the findings of this study, it is recommended that the government increase free or low-cost sports facilities in communities and towns where groups with low educational attainment gather to lower the threshold for exercise participation. Communities are advised to promote low-cost fitness programs such as square dance courses and community morning running groups, while enterprises should develop cost-effective and durable sports products to cater to the practical consumption preferences of this group.

However, this study has limitations in the single distribution of sample regions and educational levels, failure to control confounding factors such as income and occupation, and use of cross-sectional data, which makes it difficult to reveal the dynamic relationships among variables. It is suggested that future studies should expand the sample to cover low-education groups in different regions and occupations, control potential confounding factors, adopt longitudinal tracking designs to explore the causal relationships and developmental laws among exercise attitudes, exercise volume, and consumption demand, and conduct subgroup analyses to deeply investigate the heterogeneity of consumption behaviors among groups with different educational levels.

## Data Availability

The raw data supporting the conclusions of this article will be made available by the authors, without undue reservation.
